# Biomechanical and morphological stability of acellular scaffolds for tissue-engineered heart valves depends on different storage conditions

**DOI:** 10.1007/s10856-018-6106-9

**Published:** 2018-07-03

**Authors:** Piotr Wilczek, Gach Paulina, Jendryczko Karolina, Marcisz Martyna, Wilczek Grazyna, Major Roman, Mzyk Aldona, Sypien Anna, Samotus Aneta

**Affiliations:** 1 Heart Prosthesis Institute, Bioengineering Laboratory, Wolnosci 345A, 41-800 Zabrze, Poland; 2 Department of Animal Physiology and Ecotoxicology, Faculty of Biology and Environmental Protection, University of Silesia, Bankowa 9, 40-007 Katowice, Poland; 3 Institute of Metallurgy and Materials Science, Reymonta 24, 30-059 Krakow, Poland

## Abstract

Currently available bioprosthetic heart valves have been successfully used clinically; however, they have several limitations. Alternatively, tissue-engineering techniques can be used. However, there are limited data concerning the impact of storage conditions of scaffolds on their biomechanics and morphology. The aim of this study was to determine the effect of different storage conditions on the biomechanics and morphology of pulmonary valve dedicated for the acellular scaffold preparation to achieve optimal conditions to obtain stable heart valve prostheses. Scaffold can then be used for the construction of tissue-engineered heart valve, for this reason evaluation of these parameters can determine the success of the clinical application this type of bioprosthesis. Pulmonary heart valves were collected from adult porcines. Materials were divided into five groups depending on the storage conditions. Biomechanical tests were performed, both the static tensile test, and examination of viscoelastic properties. Extracellular matrix morphology was evaluated using transmission electron microscopy and immunohistochemistry. Tissue stored at 4 °C exhibited a higher modulus of elasticity than the control (native) and fresh acellular, which indicated the stiffening of the tissue and changes of the viscoelastic properties. Such changes were not observed in the radial direction. Percent strain was not significantly different in the study groups. The storage conditions affected the acellularization efficiency and tissue morphology. To the best of our knowledge, this study is the first that attributes the mechanical properties of pulmonary valve tissue to the biomechanical changes in the collagen network due to different storage conditions. Storage conditions of scaffolds for tissue-engineered heart valves may have a significant impact on the haemodynamic and clinical effects of the used bioprostheses.

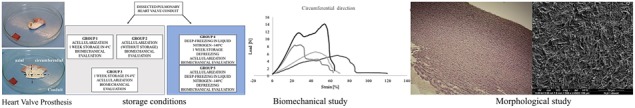

## Introduction

The number of patients with valve heart disease is increasing due to population ageing and unresolved problems of rheumatic heart disease in developing countries. In 2050, the number of people requiring heart valve replacement is expected to reach 850,000 a year [1–5]. The currently available bioprosthetic heart valves have been successfully used clinically; however, they have a number of limitations. In biological prostheses, the degenerative process and calcification strongly reduce their durability [6, 7]. Although mechanical prostheses have a longer lifespan, there is a risk of damage to blood morphotic elements that requires long-term anticoagulant treatment [8–10]. As an alternative for the creation of heart valve bioprostheses, tissue-engineering techniques can be used. Modern methods of tissue-engineering use biological tissue subjected to an acellularization procedure using enzymatic or chemical methods. Next, the acellular scaffold can be seeded with autologous cells [11–15]. The acellular scaffold should demonstrate biomechanical properties as close as possible to those of the native tissue. The tissue biomechanics may depend on various factors, such as the age of the donor and tissue source [16, 17]. In the case of tissue-engineering techniques, the acellularization method can also affect the biomechanics and morphology of the tissue [18]. The storage conditions of the scaffolds could also be an important factor that may affect the mechanical stability and morphology of the bioprosthetic heart valves. The understanding of the effect of storage conditions on the nature of changes in the scaffold morphology and biomechanics is crucial because of its important clinical significance, both for the postoperative and long-term results. This effect determines the possibility of maintaining the correct haemodynamics of the bioprosthesis [19]. However, due to limited sources of fresh tissue, the key is to develop the criteria for scaffold storage, thereby affecting the experimental and clinical significance of the process of bioprosthesis preparation. Moreover, from the clinical point of view, it is important to fulfil the “off-the-shelf-organs” criteria to increase the availability of a specific type and size of heart valve scaffold, depending on current requirements, for the given patients. Commonly used methods of tissue storage include fresh storage at 4 °C or deep-freezing in liquid nitrogen. Although, during tissue storage, dedicated media are used that help to maintain unchanged morphological and biomechanical properties of the tissue [20, 21], it is known that cold storage may induce cellular and extracellular matrix damage. Storage of the tissue in liquid nitrogen causes a risk of damage to collagen and elastin fibres and smooth muscle cells. This damage is mainly related to water redistribution and ice crystal production, which cause mechanical alteration to the ECM and cells [22, 23]. In turn, the disadvantages of 4 °C tissue storage (homovital) are mainly related to the limited duration of use; at the time of implantation, the valves are non-viable, ultimately affecting the valve durability [24]. Although bioengineered heart valves are subjected to the acellularization process, the cell viability does not appear to be narrowly critical; however, the storage conditions can affect the mechanical properties of the tissue, causing a preliminary alteration in morphology that affects the quality of the scaffolds dedicated for bioprosthesis creation. It is known that necrotic cells release proinflammatory “damage‐associated molecular patterns” (DAMPs), which trigger cell injury and induce inflammatory responses that promote tissue fibrosis in vitro and in vivo [25]. The inflammatory response occurs in the absence of pathogens, defined as “sterile inflammation”, and may induce tissue injury in vitro. As postulated by Matzinger, the immune system detects both nonself molecules and molecules not ordinarily found in extracellular fluids in the absence of cell death, damage, or stress [26]. The necrotic myocardial cells (NMCs) release DAMPs, which provoke fibroblast activation in vitro, as well as myocardial inflammation and fibrosis in vivo. Various mechanisms responsible for ECM damage make their identification critical for the bioengineering of heart valve. To date, biomechanical studies have focused mainly on the impact of acellularization methods on the condition of the scaffolds. In our study, we tested different conditions of tissue storage, allowing for continuous availability of tissue scaffolds used in valvular bioprosthesis preparation. The present study attempted to determine the optimal conditions for tissue preservation such that it does not significantly affect the morphology and biomechanics of pulmonary valve scaffold, which can be next used for the bioprosthesis preparation based on the tissue-engineered techniques. Our results indicate that the storage conditions may be an important determinant of long-term bioprosthesis durability.

## Materials and methods

### Sample collection

**Fig. 1:**
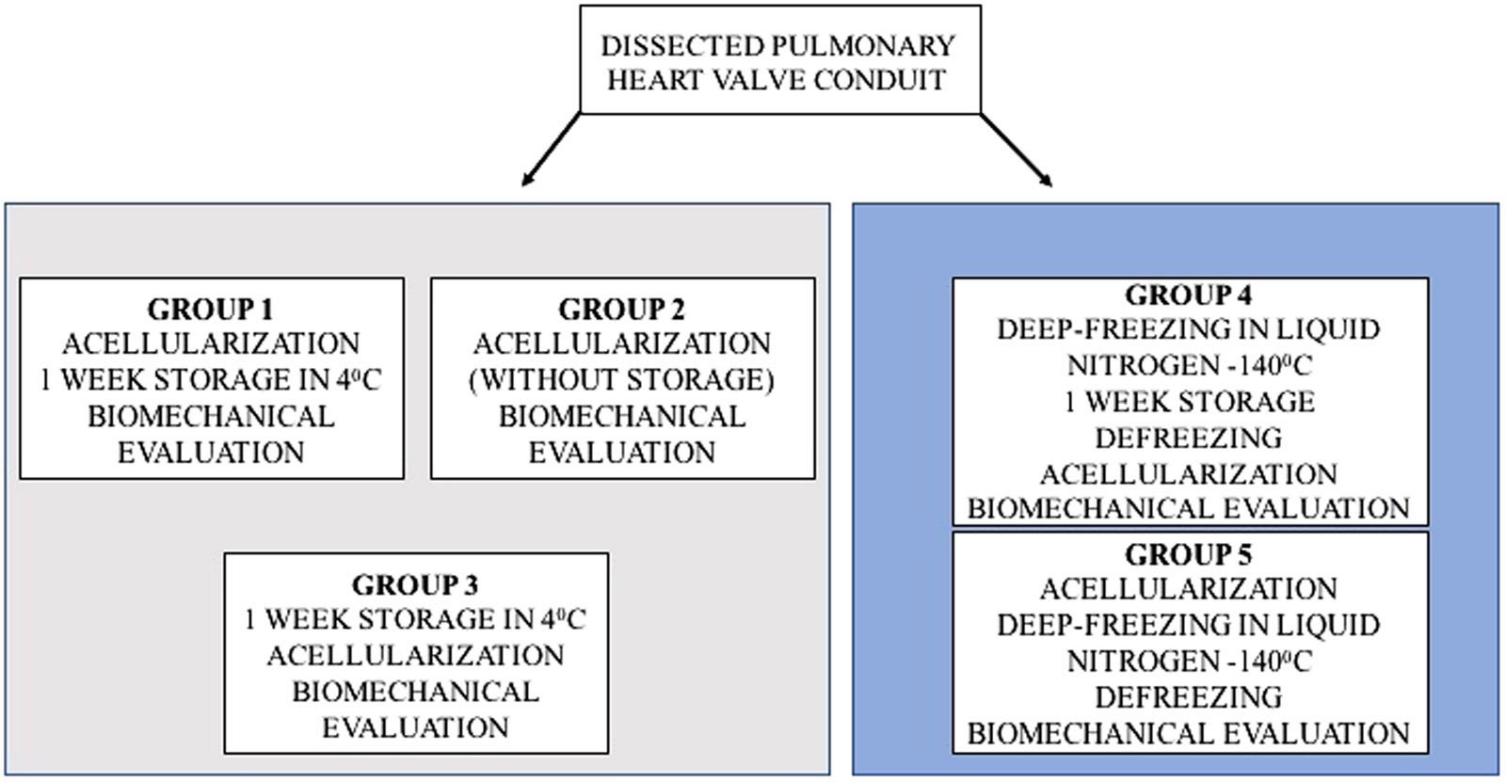
Division of the evaluated study groups depending on the temperature and time of tissue storage

Porcine hearts were collected from adult animals with a weight ranging from 70 kg to 80 kg. Explanted hearts were placed in sterile containers in Ringer solution (Solutio Ringeri; FRESENIUS KABI) and were transported on ice to the laboratory in less than 1 h. From each heart, the pulmonary valve was dissected. The samples were rinsed with Ringer solution for blood removal and were incubated for 24 h in 4 °C in an antibiotic bath containing 1% penicillin/streptomycin (Gibco), 0.1% Ciprinol (Krka) and 0.2% Mycomax (Zentiva) dissolved in 1000 ml of Ringer solution. The samples were divided into five groups according to the diagram below (Fig. 1).

For Group 1, the pulmonary valve conduit was collected and was subsequently incubated for 24 h in an antibiotic bath. Next, acellularization was performed followed by storage of the tissue for one week at 4 °C and performance of biomechanical tests. For Group 2, after 24 h in the antibiotic bath, acellularization was performed, and the biomechanical properties were evaluated. For Group 3, the tissue was stored for one week at 4 °C, and the acellularization procedure and biomechanical tests were performed. For Group 4, the tissue was stored in liquid nitrogen at −140 °C for 1 week and was subsequently thawed followed by performance of the acellularization procedure and biomechanical tests. For Group 5, the acellularization procedure was performed, and the tissue was later stored in liquid nitrogen at −140 °C for 1 week followed by thawing and performance of biomechanical tests. As a control the native tissue, directly after antibiotic bath was used. All of the samples underwent biomechanical testing including the static tensile test, examination of viscoelastic properties, and evaluation of extracellular matrix morphology using transmission electron microscopy (TEM). The estimation of elastin, fibronectin, collagen I, collagen IV, elastic tissue fibres—Verhoeff’s Van Gieson (EVG) and Van Kossa to quantify mineralisation in tissue sections, as well as histological staining, haematoxylin and eosin (H&E) Masson trichrome for the estimation of the changes in connective tissue.

### Acellularization

The valves were rinsed in phosphate-buffered saline (PBS; GIBCO) and were placed in trypsin/EDTA (1 × 0.5 g/l porcine trypsin and 0.2 g/l EDTA in Hank’s Balanced Salt Solution; GIBCO) for 48 h with shaking, and trypsin was replaced every 24 h. After 48 h of enzymatic digestion, the tissue was placed in 0.5% sodium dodecyl sulphate (SDS; Sigma) for 15 min. To remove residual enzymes and detergent, the heart valve samples were washed in PBS several times for 1 h at room temperature with shaking.

### Liquid nitrogen freezing and thawing

The freezing medium consisted of RPMI 1640 medium, fetal bovine serum (FBS) and DMSO as a cryoprotectant at a ratio of 8:1:1. Before freezing, the tissue samples were packed into three sterile bags and a cardboard box. Tissue freezing was carried out gradually at the rate of 1 °C/min, up to (-) 40 °C, and then the tissue was transferred to the Dewars and stored in liquid nitrogen vapour at (-) 170 °C. The thawing procedure involves placing the sample for 20 min at room temperature, and thereafter transferring the samples to cold water until the ice melted. To remove the DMSO, the samples were placed first in 100 ml of freezing solution, the thawing medium (RPMI, FBS in ratio 9: 1) was added, and then the tissue was washed in the following volumes: 33 ml of thawing medium for 1 min, 66 ml of thawing medium for 1 min, and 99 ml of thawing medium for 1 min.

### Biomechanical examination

**Fig. 2:**
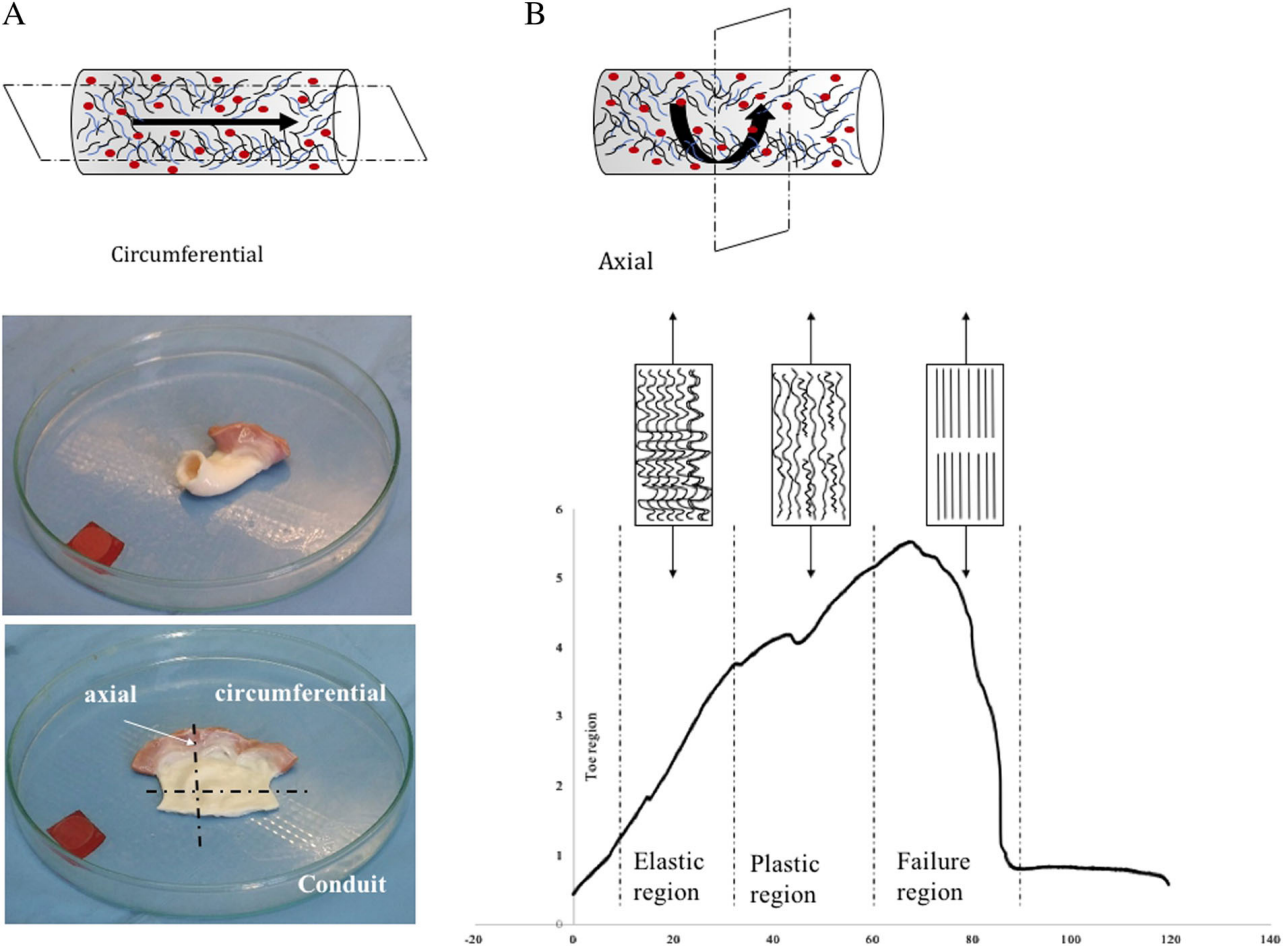
**a**, **b** Orientation of the samples for biomechanics. Valve fragments were dissected in two orientations, circumferential (sens of blood flow) and axial (perpendicular to blood flow). **b** Typical stress–strain curve for destructive tensile tensing of collagen tissue. Collagen fibril straightening and failure related to different regions of the stress–strain curve

Uniaxial tensile tests were performed using a universal testing machine (Tytron 250 Microforce Testing System MTS, MTS Systems Corporation, Eden Prairie, MN, US) with a 250-N force transducer. The percent strain was measured with a video extensometer (Video Extensometr NG version 5.15.5.0 Messphysik equipped with the Blue FOX camera with the resolution of 5 megapixel). For the proper analysis of the percent strain, two black and white targets placed on the sample were used. For good contrast, the sample was placed on a black background. Before each test video, the extensometer was calibrated using patterns provided by the manufacturer. The use of the targets on the sample avoided measuring errors caused by the clamps and allowed the measurement of the real elongation. The specimen was cut with special devices to form a dumbbell-shaped sample between the holder’s area. The dumbbell shape minimised the influence of Poisson effects on strain measurements in the longitudinal direction of the sample. The sample was placed in the holder and wrapped with the gauze to avoid slipping. The use of the gauze protects the tissue against crushing and non-specific rupture of the tissue in the holders. Before initiating the test, the thickness of the sample was measured with a caliper with the resolution of 0.02 mm, and the width was measured with a video extensometer. The thickness and width were used as the input data to calculate the area of the sample automatically to obtain the value of the elastic modulus. Before the test was started, all of the samples were preloaded to the 0.5 N force. The tissue was loaded at 20 mm/min until the sample broke. The data from the displacement and force sensor between the targets on the sample were recorded online on a personal computer. Elastic moduli were obtained from the slope of the initial linear section of the stress–strain curve (tgu). The analysis was performed up to 30% strain in the mechanical tests. At this value, the corresponding stress is equal to or higher than the maximal stress experienced by the valve leaflets in situ. Above this value, there was an increased possibility of sample slippage and crack formation on the edges. The strain was calculated from the extension data according to the following formula:

$$\varepsilon = \frac{{{\mathrm t}{\mathrm e}{\mathrm n}{\mathrm s}{\mathrm i}{\mathrm l}{\mathrm e}\,{\mathrm s}{\mathrm t}{\mathrm r}{\mathrm e}{\mathrm s}{\mathrm s}}}{{{\mathrm t}{\mathrm e}{\mathrm n}{\mathrm s}{\mathrm i}{\mathrm l}{\mathrm e}\,{\mathrm s}{\mathrm t}{\mathrm r}{\mathrm a}{\mathrm i}{\mathrm n}}} = \frac{\sigma }{\varepsilon } = \frac{{F/A_0}}{{\Delta L/L_0}} = \frac{{FL_0}}{{A_0\Delta L}}$$

where *E* is the Young’s modulus (modulus of elasticity), *F* is the force exerted on an object under tension, *A*_0_ is the original cross-sectional area through which the force is applied, DL is the amount by which the length of the object changes, *L*_0_ is the original length of the object after preloaded to the value 0.5. Additionally, the energy to break was measured and is defined as the energy delivered to the sample required to break the sample. The peak strain is defined as the strain of the sample at the maximum force. The peak load is defined as the maximum force until the tissue breaks, and the peak stress indicates the maximum force per area. Additionally, the value of slope 1, which is the initial region of the stress–strain curve and slope 2, which indicates the stiff region of the stress–strain curve, was calculated. To test the viscoelastic response, the hysteresis test was performed. Before the test, the sample was preloaded to 1 N, loaded onto the 10% strain, held at 60 s, and retracted to the base value. The following values were calculated from the test: modulus of loading, energy loading, and energy unloading. All of the biomechanical parameters were tested in the axial and circumferential directions (Fig. 2a).

The acquisition and analysis of the tested parameter were performed using Test Works software (MTS Systems Corporation, Eden Prairie, MN, USA).

### Histology and immunohistochemistry

Acellular tissue was subjected to histological evaluation. Specimens for microscopic examinations were fixed in 6% buffered neutralised formaldehyde. The specimens were routinely processed and embedded in paraffin, and the 5-μm slides were cut using a rotary microtome. The tissue was stain with haematoxylin and eosin (H&E) to evaluate cell removal effectiveness; additionally, Masson trichrome for connective tissue was performed. The slides were viewed in visible light using a Reihert Polyvar 2 microscope. For immunohistochemistry directly after collection, the tissue was placed in Tissue Freezing Medium and stored for 12 h at room temperature, and then the sample was stored in a freezer (−70 °C) until cutting. After cutting using the Thermo Shandon Cryotome FSE, frozen sections with a thickness of 5 mm were subjected to incubation with the appropriate monoclonal antibodies. For immunohistochemistry, EnVision AP chromogen (New Fuchsin Substrate System/Fast Red, DAKO) was used according to the manufacturer’s instructions. The following stainings were performed to quantify mineralisation in the tissue sections: elastin, fibronectin, collagen I, collagen IV, Elastic Tissue Fibres–Verhoeff’s Van Gieson (EVG) and Van Kossa.

### Scanning electron microscopy

Fractured characterisation of the samples was performed using scanning electron microscopy (SEM) (FEI Quanta 3D FEG equipped with the Trident energy-dispersive X-ray spectrometer produced by EDAX in the Accredited Testing Laboratories-IMMS, PAS). Samples were coated with 10 ÷ 150 nm gold/palladium before microstructural studies (vacuum, 5 × 10^6–5^ mbar; deposition atmosphere, 1 × 10^−2^ mbar; 50 s; 40 mA; distance, 50 mm). The structural research was conducted at accelerating voltages of 5 kV, a working distance of 7.5 mm in secondary electrons (SEs) and a variable vacuum mode.

### Statistical analysis

The results were analysed statistically using STATISTICA v. 10.0 software. All assays were based on 8–10 samples, performed in duplicate. The mean values and standard deviations were calculated. The significance of differences between the experimental groups in the levels of the analysed parameters in relation to the tissue storage methods was assessed using analysis of variance (ANOVA/MANOVA) with the Tukey’s test for unequal sample size at *P* < 0.05. The homogeneity of variance was tested by Levene’s test.

## Results

### Biomechanical examination

Both factors within the distinguished grouping variables, namely, the type of valve and direction of cutting, were significantly affected and differentiated groups subjected to similar treatment, except Slope 1 and Slope 2. The strongest effect was produced by the type of valve, as manifested by changes in all the analysed parameters. The weakest effect was induced by direction cutting, which caused differences in the level of the modulus and peak load, irrespective of the valve type (Tables 1 and 2).

**Table 1:** Analysis of variance (ANOVA/MANOVA) for selected parameters: Modulus, Strain at peak, and Energy to break from experimental groups (with type of valve and direction cutting as categorical factors)

Source of variation	Modulus			Strain at peak			Energy to break		
	Ms Effect	*F*	*P*	Ms Effect	*F*	*P*	Ms Effect	*F*	*P*
Type of valve	1.3	7.5	< 0.001	233.4	2.8	0.03	1.74E + 07	5.1	0.001
Direction	2.2	12.6	< 0.001	367.8	4.4	0.04	1.62E + 07	4.8	0.04
Type of valve/ direction	7.5	8.5	< 0.001	316.5	3.8	0.005	2.55E + 07	7.5	< 0.001

**Table 2:** Analysis of variance (ANOVA/MANOVA) for selected parameters: Peak load, Peak stress, Slope 1, and Slope 2 from experimental groups (with type of valve and direction cutting as categorical factors)

Source of variation	Peak load			Peak stress			Slope 1			Slope 2		
	Ms Effect	*F*	*P*	Ms Effect	*F*	*P*	Ms Effect	*F*	*P*	Ms Effect	*F*	*P*
Type of valve	46.2	8.5	< 0.001	0.2	3.3	0.011	1,222,991	10.6	< 0.001	1,856,718	7.2	< 0.001
Direction	51.0	9.4	0.004	0.2	2.9	0.10	15,514	0.1	0.7	44,742	0.2	0.7
Type of valve/direction	162.1	29.9	< 0.001	0.5	8.2	< 0.001	81,549	0.7	0.6	133,585	0.5	0.8

**Fig. 3:**
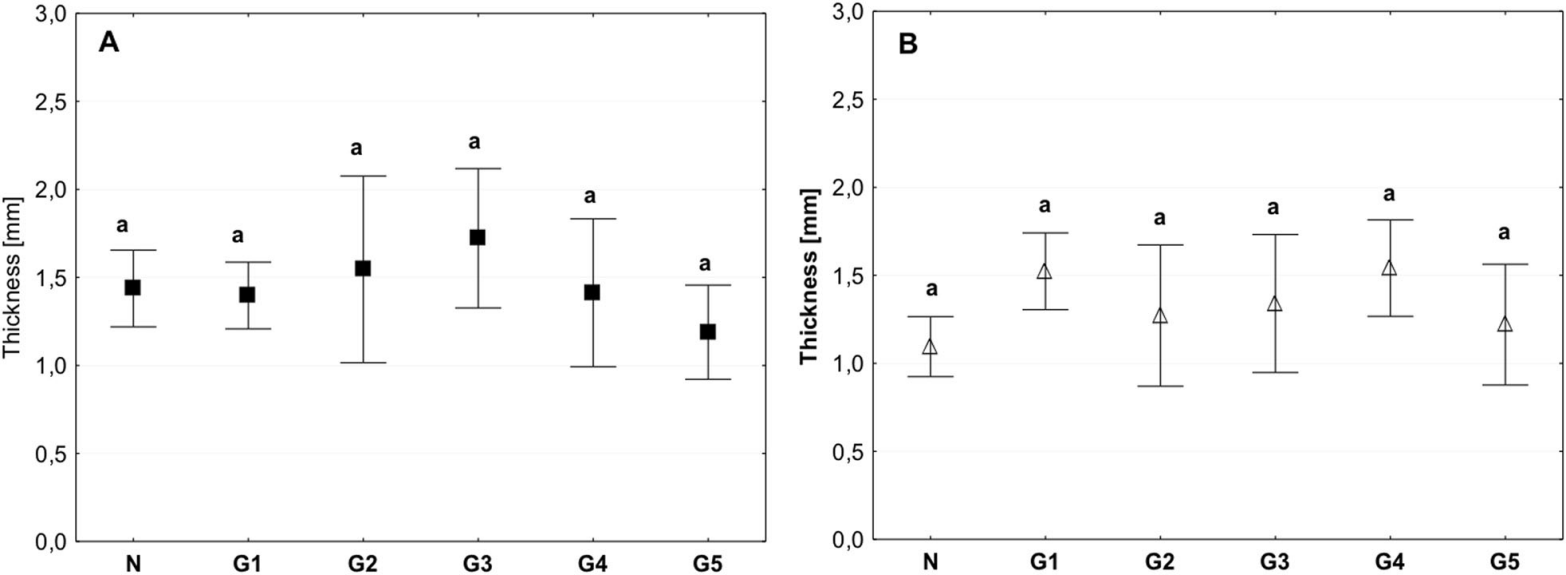
Mean value of tissue thickness in the circumferential (**a**) and axial directions (**b**) (average ± SD) in the study groups. The same letters (a) indicate homogeneous groups within individual valves (Tukey’s test, *P* < 0.05; *N* = 8–10)

The examined tissue indicated homogeneity in terms of morphology, the thickness of the tissue was similar for all groups, and the value was approximately 1.5 mm (Fig. 3a, b).

**Fig. 4:**
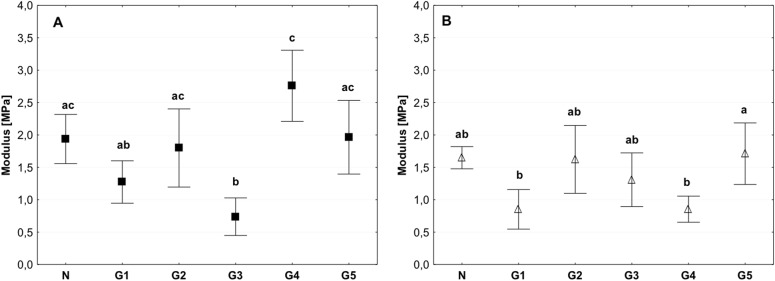
Mean value of the modulus of elasticity in the circumferential (**a**) and axial directions (**b**) (average ± SD) in the study groups. The same letters (a, b, c) indicate homogeneous groups within individual valves (Tukey’s test, *P* < 0.05; *N* = 8–10)

The value of the modulus of elasticity was lower in the group of tissues stored at 4 °C compared than in the control and deep-freezing tissue. Significant differences were observed between the control and G3 groups for which this value was approximately 0.5 Mpa; however, in the control, the value was approximately 2 Mpa. Statistically significant differences were observed among the G3, G4, and G5 groups (Fig. 4a, b). The modulus of elasticity for the tissue from G2 group was comparable to the control both for the circumferential and axial direction tests (Fig. 4a, b).

**Fig. 5:**
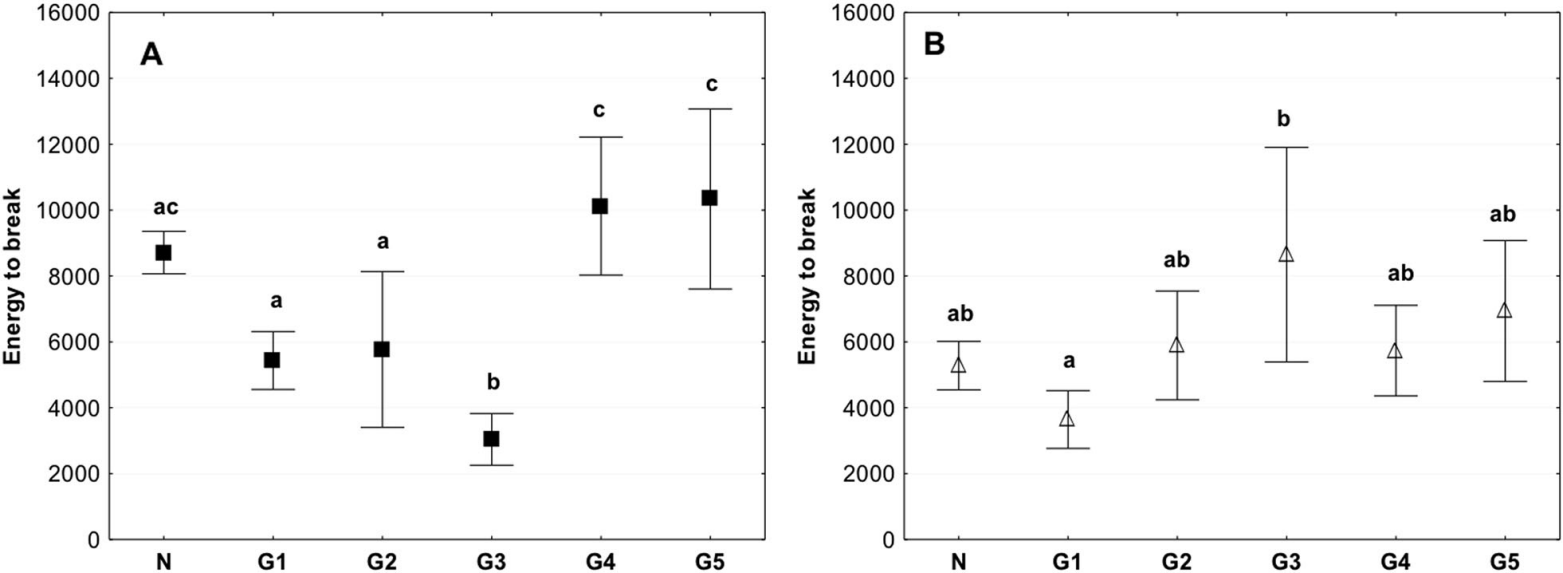
Mean value of energy to break in circumferential (**a**) and axial direction (**b**) (average ± SD) in study groups. The same letters (a, b, c) indicate homogeneous groups within individual valves (Tukey’s test, *P* < 0.05; *N* = 8–10)

The estimation of Energy to break showed that the value obtained for the G3 group was significantly lower compared to other groups. The value was <3000 J/m^2^, while in the control group, it was 8000 J/m^2^ and for the G4 and G5 group the energy to break reached the value of more than 10,000 J/m^2.^ (Fig. 5a). While the Energy to break measured in the axial direction demonstrate greater homogeneity (Fig. 5b). The Energy to Break for the G2 group was slightly lower than for the control in the circumferential direction, in the axial direction it achieved values similar to those in the control. Both in the circumferential and axial direction the value of Energy to Break was significantly lower than noted for the G4 and G5 group (Fig. 5a, b).

**Fig. 6:**
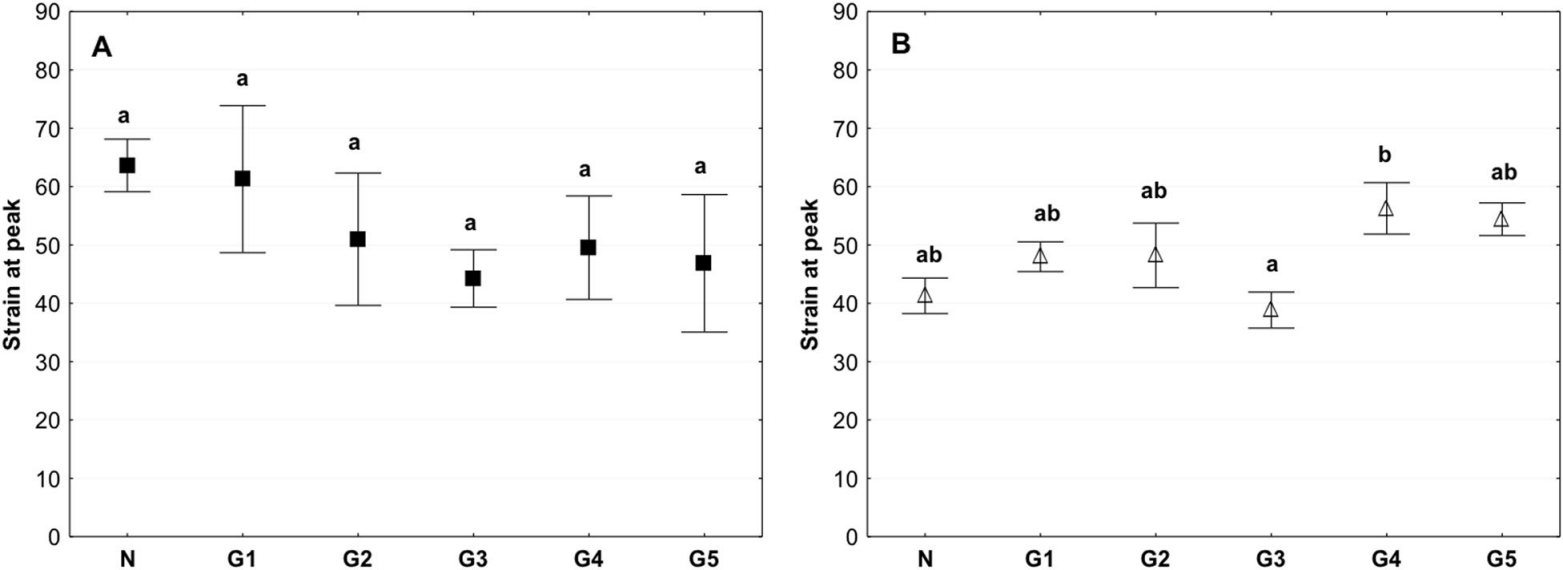
Mean value of the peak strain in the circumferential (**a**) and axial directions (**b**) (average ± SD) in the study groups. The same letters (a, b) indicate homogeneous groups within individual valves (Tukey’s test, *P* < 0.05; *N* = 8–10)

There were no significant differences in the peak strain between the groups in the samples investigated in the circumferential direction. In the samples examined in the axial direction, the only differences were observed between the G3 and G4 groups (Fig. 6b). No differences were observed between the G2 group and control (Fig. 6a, b).

**Fig. 7:**
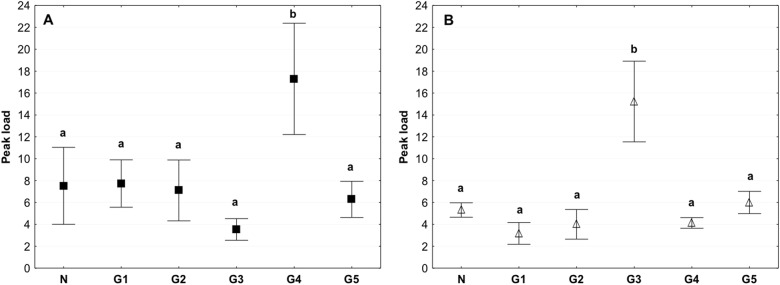
Mean value of the peak load in the circumferential (**a**) and axial directions (**b**) (average ± SD) in the study groups. The same letters (a, b) indicate homogeneous groups within individual valves (Tukey’s test, *P* < 0.05; *N* = 8–10 per group)

The peak load value, which indicates the maximum force until the tissue breaks, differed significantly in the G4 group in which the value was higher than that in other groups. In the circumferential direction, the peak value reached approximately 17 N. In the axial direction, the value reached approximately 15 N, and it was almost two times higher than that in the other groups (Fig. 7a, b). The Peak load value for the G2 group was about 8 N for tests performed in the circumferential direction, and it was similar than that for the control, in turn for tests performed in the axial direction this value was 4 N and was slightly lower than than in the control group (Fig. 7a, b).

**Fig. 8:**
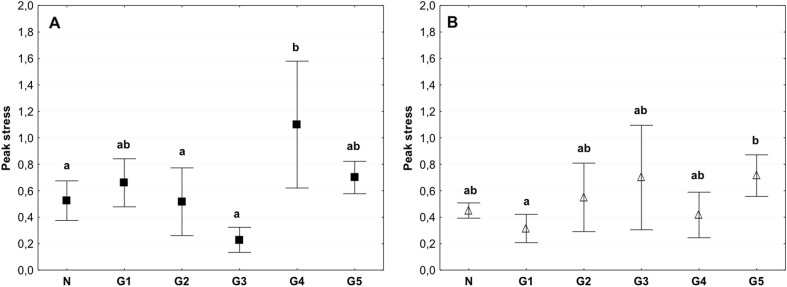
Mean value of the peak stress in the circumferential (**a**) and axial directions (**b**) (average ± SD) in the study groups. The same letters (a, b) indicate homogeneous groups within individual valves (Tukey’s test, *P* < 0.05; *N* = 8–10)

A similar trend was recorded for the peak stress for samples tested in the circumferential direction (Fig. 8a).

**Fig. 9:**
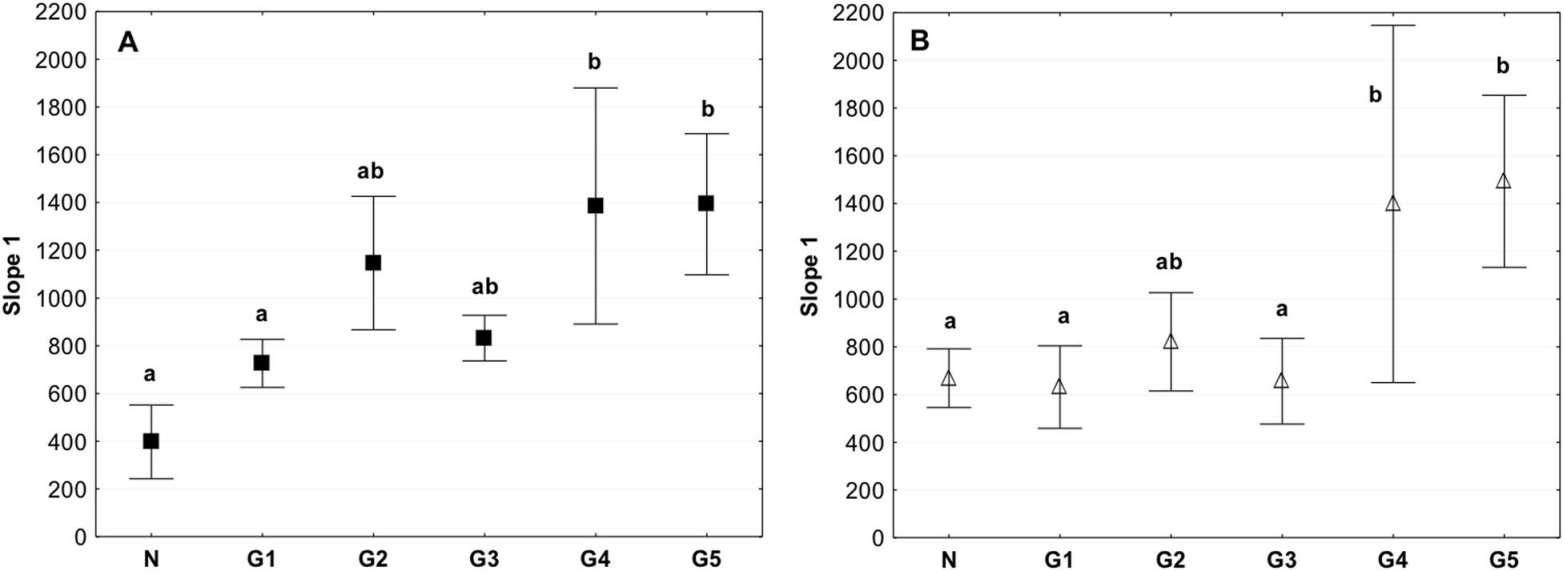
Mean value of Slope 1 in the circumferential (**a**) and axial directions (**b**) (average ± SD) in the study groups. The same letters (a, b) indicate homogeneous groups within individual valves (Tukey’s test, *P* < 0.05; *N* = 8–10)

The slope 1 value for the tested storage conditions in the circumferential and axial directions indicated a significant increase in the value in the G4 and G5 groups stored in liquid nitrogen (Fig. 9). Both for the tests performed in the circumferential and axial direction the slope 1 value for the G2 group were higher than in the control, but at the same time the value of slope 1 was lower than for the groups G4 and G5 (Fig. 9a, b).

**Fig. 10:**
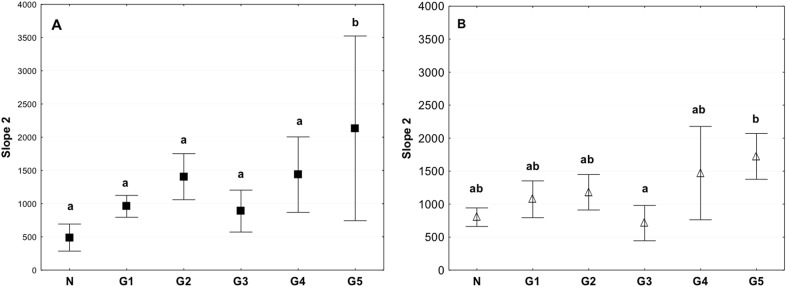
Mean value of Slope 2 in the circumferential (**a**) and axial (**b**) directions (average ± SD) in the study groups. The same letters (a, b) indicate homogeneous groups within individual valves (Tukey’s test, *P* < 0.05; *N* = 8–10)

The control and 4 °C stored tissue had significantly lower values of slope 2 than tissues in the G5 group that were subjected to the acellularization procedure, followed by storage in liquid nitrogen at −140 °C for 1 week, and thawing and performance of biomechanical tests (Fig. 10). Similar to the slope 1 in the tests performed in the circumferential and axial direction the value obtained for the G2 group were higher than in the control, but at the same time lower than for the groups G4 and G5 (Fig. 10a, b).

### Histology and immunohistochemistry

**Fig. 11:**
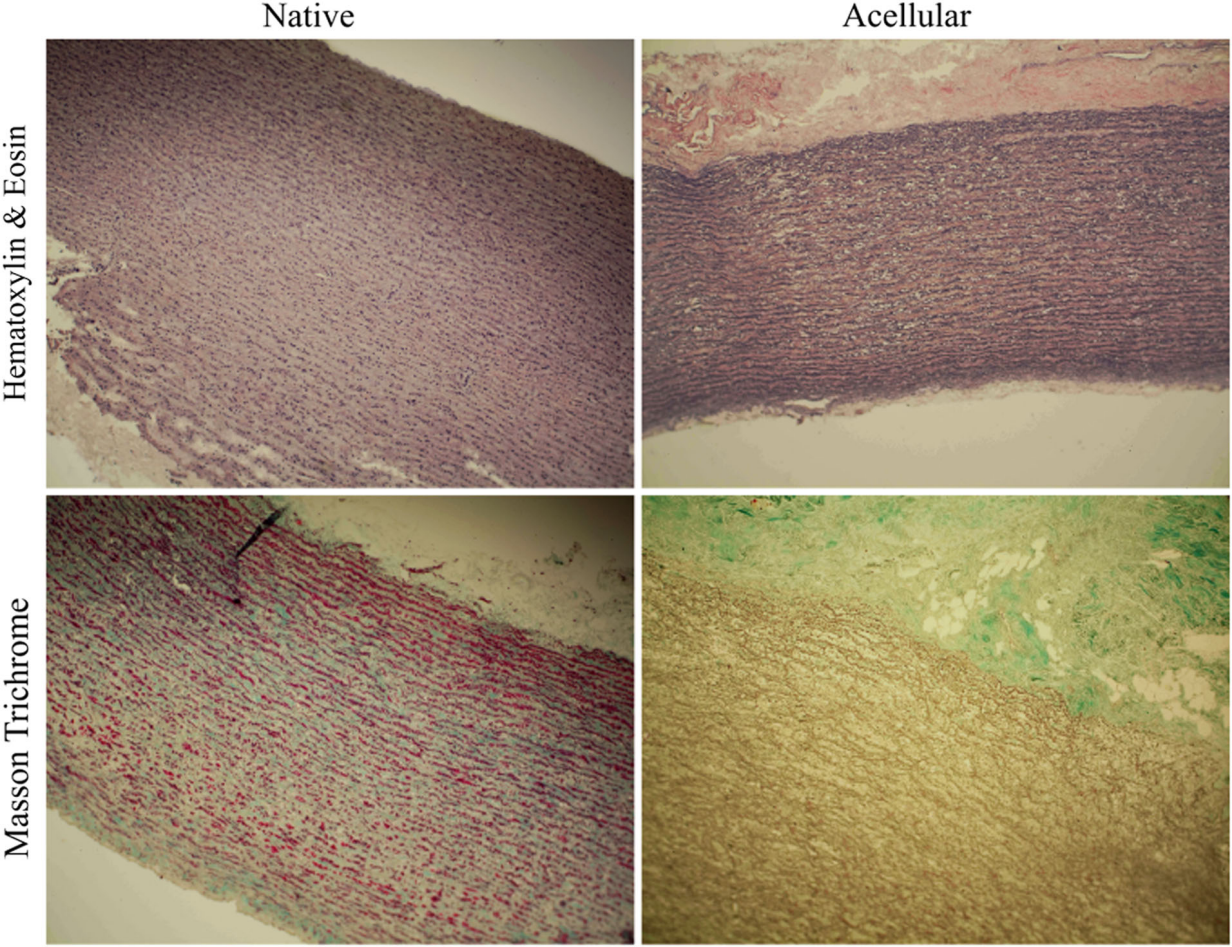
Representative image of a control tissue sample and after the acellularization procedure—Haematoxylin and Eosin staining. Cross-section of the control and acellular tissue samples—Trichrome Masson staining. The red colour represents muscle fibres, and the green colour represents collagen. After acellularization, the muscle cells were removed from the tissue, and the reduction of green colouration (collagen) was observed in parallel (colour figure online)

All of the examined tissue indicated normal tissue morphology and cellularity. After acellularization, nearly complete removal of the cells was observed, and the extracellular matrix structure was maintained. However, in the case of acellular tissue, reduced colouration of Trichrome Masson staining was observed, possibly indicating quantitative and qualitative changes within the collagen (Fig. 11).

**Fig. 12:**
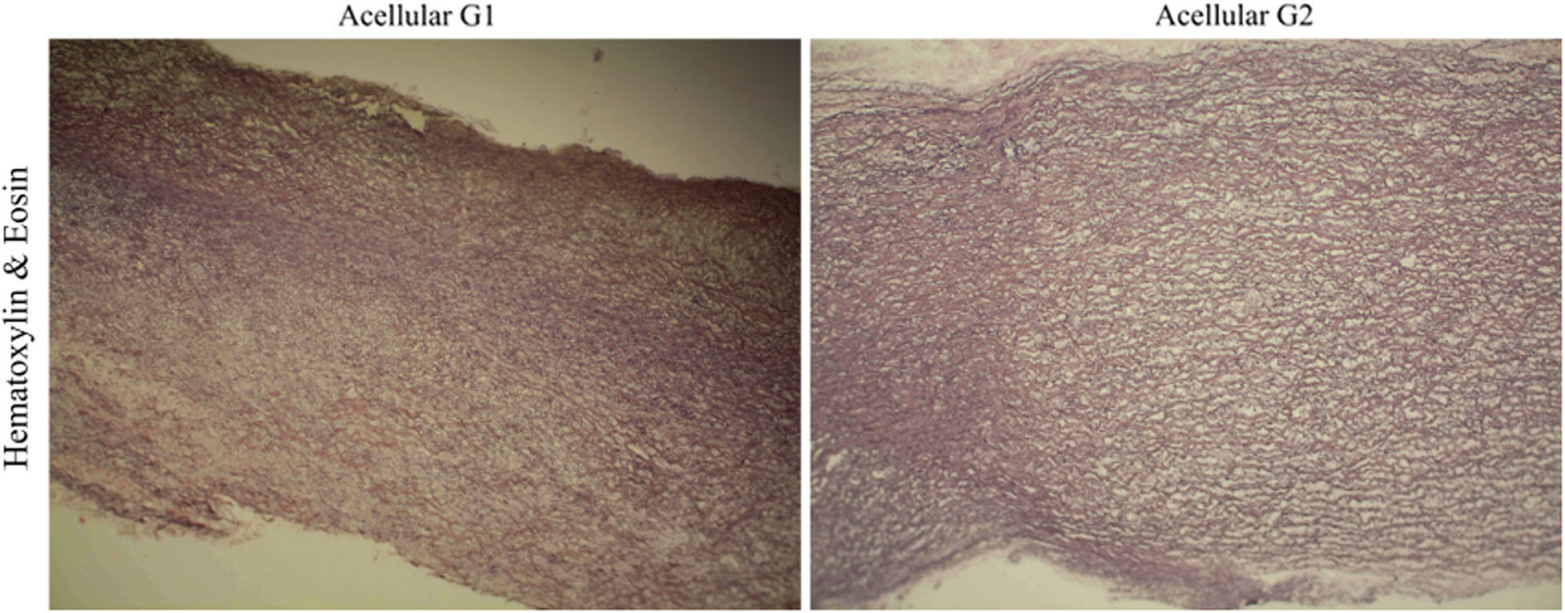
Cross-section of the acellular tissue sample derived from the G1 group (**a**) and G2 group (**b**). The haematoxylin and eosin staining indicated that the tissue in Group 2 has a more relaxed structure, with visible interfibrillar spaces

Greater compaction of the ECM after acellularization was observed within the study groups, except for the G2 group for which greater relaxation and interfibrillar spaces were observed (Fig. 12).

**Fig. 13:**
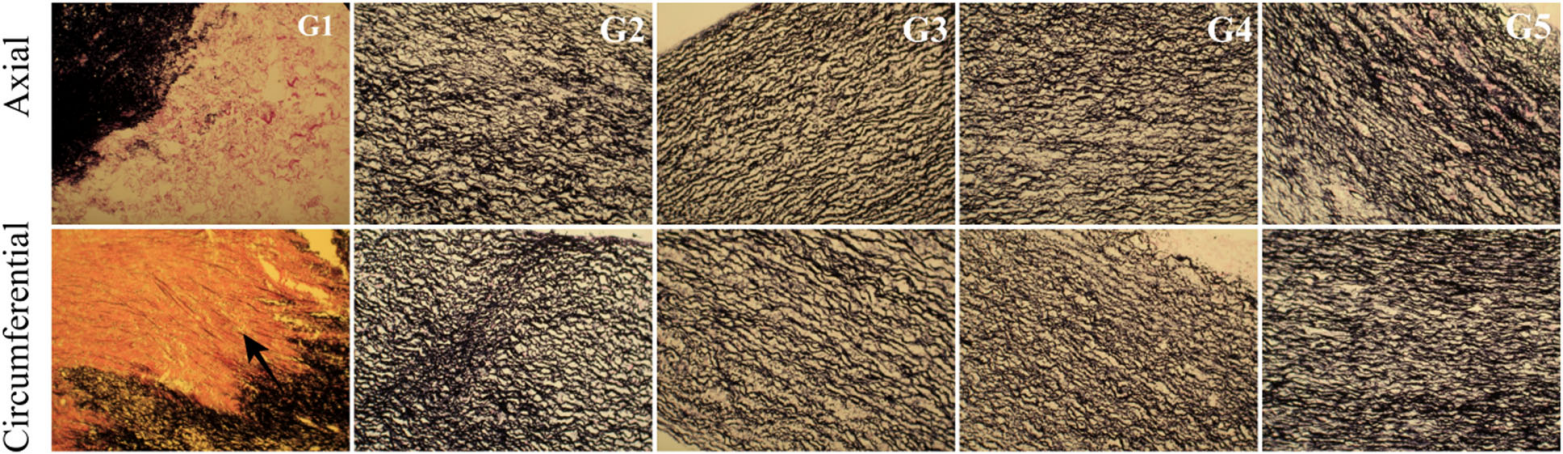
Cross-section of the acellular tissue sample prepared in the axial and circumferential directions, derived from the investigated group. EVG staining in particular investigated groups

EVG staining detects elastin fibres in histological specimens that are stained black. No significant difference in colouration was observed within the study groups, indicating a similar content and alignment of elastin fibres in the examined tissues (Fig. 13).

**Fig. 14:**
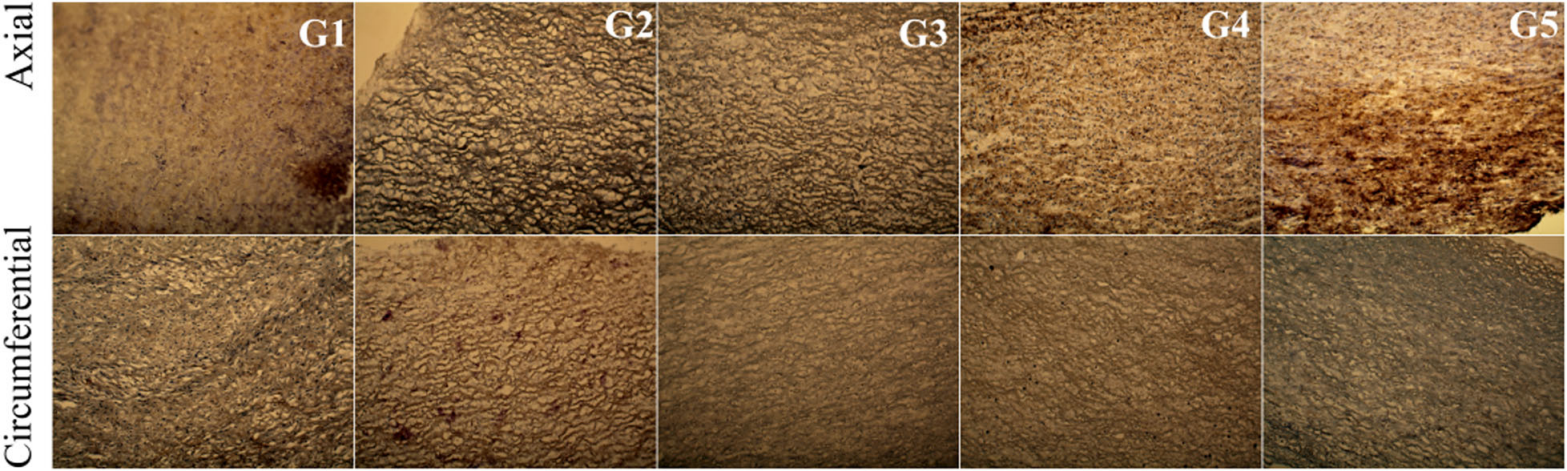
Cross-section of the acellular tissue sample prepared in the axial and circumferential directions, derived from the investigated group. Fibronectin staining in particular investigated groups

In the G2 group, in which the tissue was incubated in an antibiotic bath for 24 h, followed by acellularization and testing of biomechanical properties, larger interfibrillar spaces were observed, with similar colouration compared with the other groups. For tissues in the groups G4 and G5, lower effectiveness of the acellularization process was observed, particularly in the tissue sections used in the axial direction. Additionally, in G5, in the axial direction, more intense fibronectin staining was noted (Fig. 14).

**Fig. 15:**
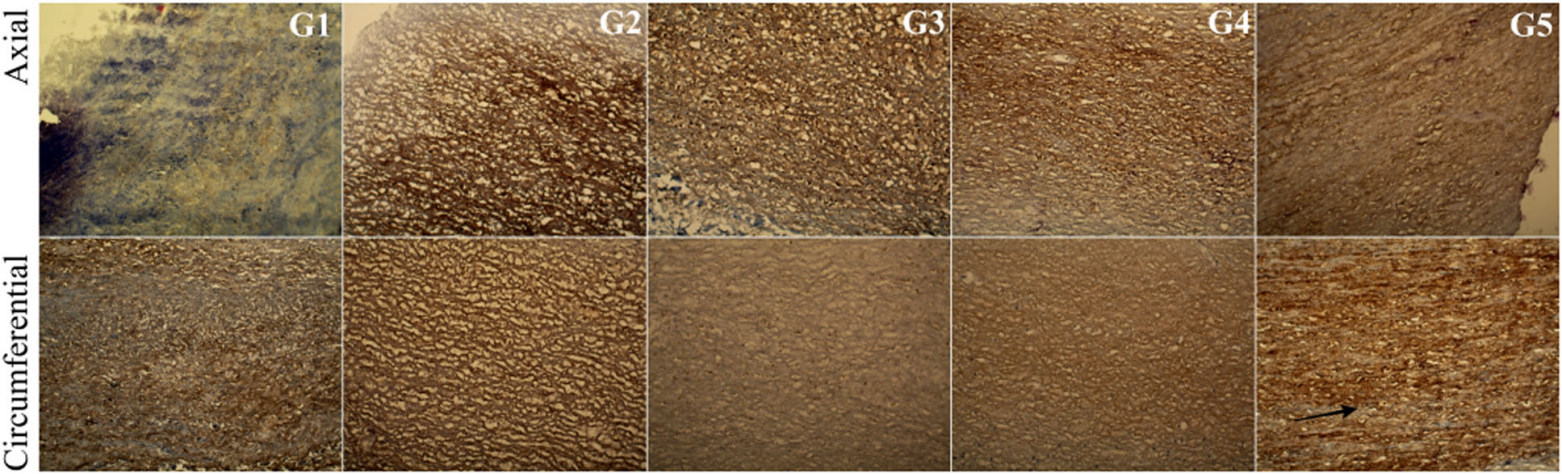
Cross-section of the acellular tissue sample prepared in the axial and circumferential directions, derived from the investigated group. Collagen IV staining in particular investigated groups

**Fig. 16:**
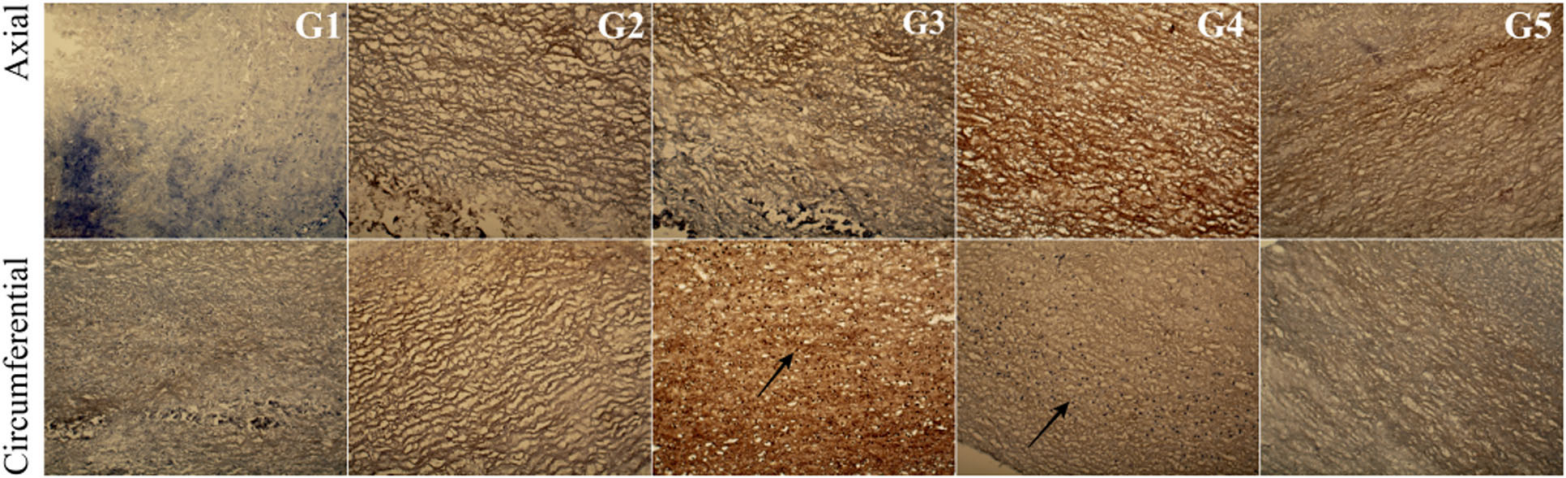
Cross-section of the acellular tissue sample prepared in the axial and circumferential directions, derived from the investigated group. Collagen I staining in particular investigated groups

**Fig. 17:**
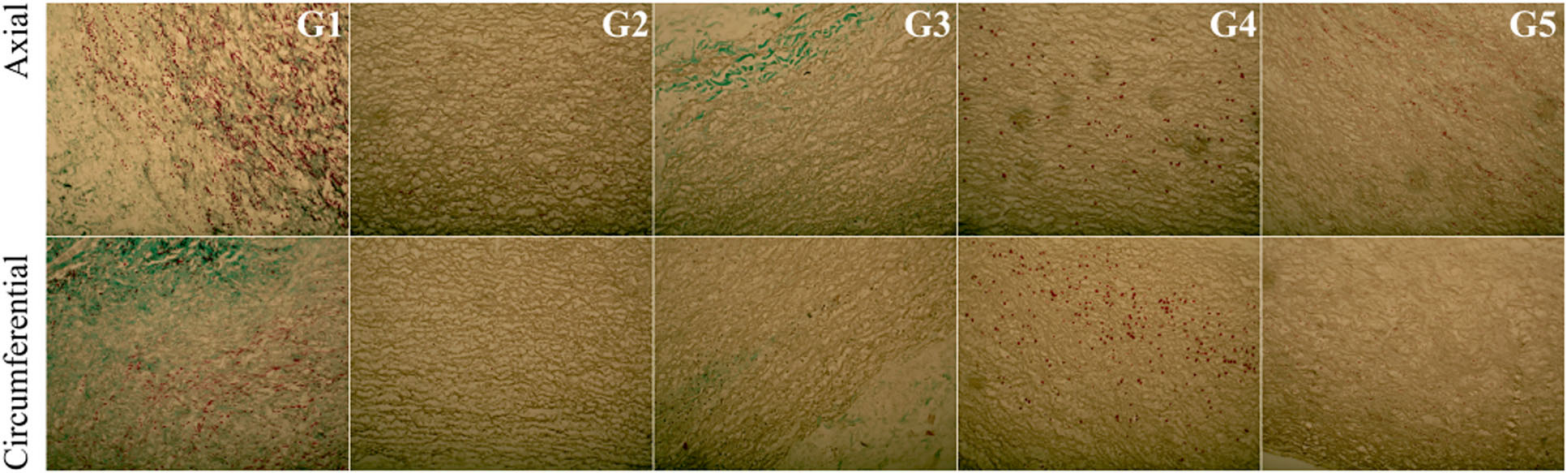
Cross-section of the acellular tissue sample prepared in the axial and circumferential directions, derived from the investigated group. Trichrome Masson staining in particular investigated groups

For collagen IV, collagen I, and Trichrome Masson staining (Figs. 15, 16, 17), similar trends were observed. The observed differences were more related to the structure, and not with the staining intensity. For the tissue subjected to deep-freezing, the tissue structure was more compacted. Particularly, in the G4 group, lower effectiveness of acellularization was observed that could be related to the structural differences.

### Scanning electron microscopy

**Fig. 18:**
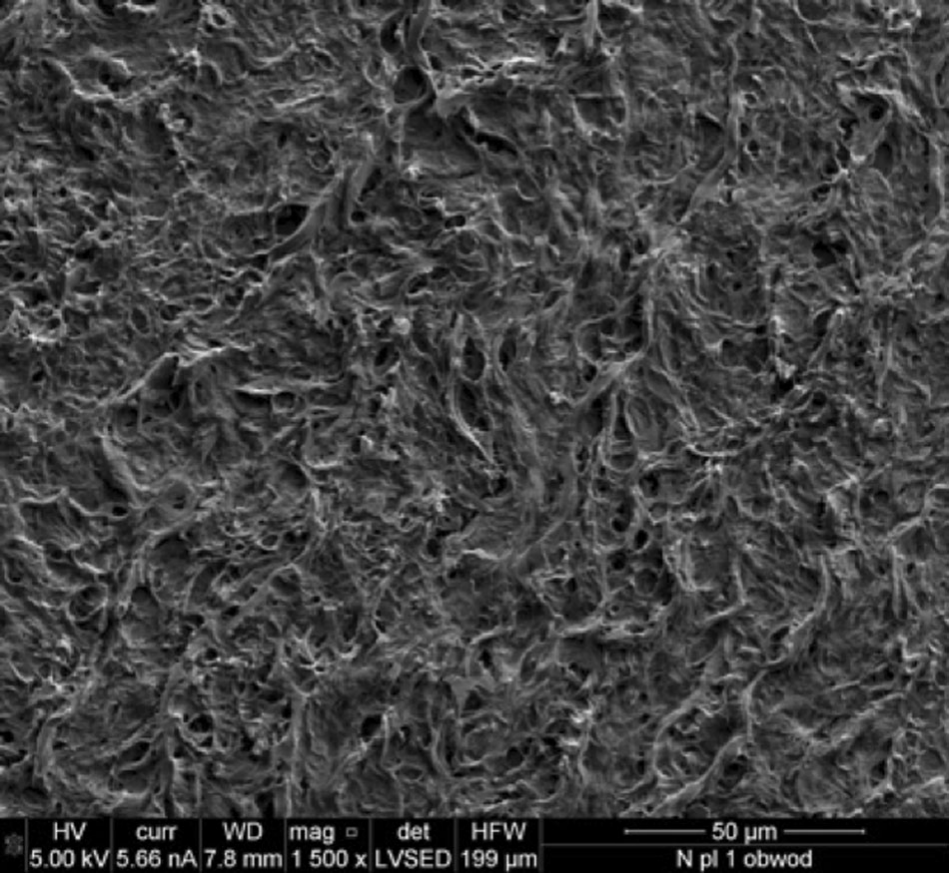
Electron microscopy section of a control pulmonary valve conduit

All the examined samples indicated normal tissue morphology with proper stitching and arrangement of extracellular matrix proteins (Fig. 18).

**Fig. 19:**
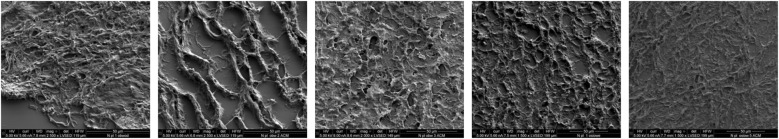
Electron microscopy section of the pulmonary valve conduit after acellularization in the study group

The studies using the electron microscopy technique (SEM) indicated that, after acellularization, almost all the cells were removed from the tissue in all the examined groups. Studies using electron microscopy showed that tissues from groups stored at 4 °C exhibited a more relaxed structure of ECM with larger interfibrillar spaces. By contrast, tissues derived from groups stored in liquid nitrogen had a slightly more compensated structure (Fig. 19).

## Discussion

Research related to tissue-engineering of heart valves has been performed for several years. However, the transfer of the research to clinical applications is very difficult. The cause may be the lack of interlaboratory standardisation of the procedures of preparation and evaluation of tissues intended for use in heart valve bioengineering. This standardisation applies, among other techniques, to the methods of tissue acellularization and storage conditions of the scaffold. These factors are very important because the success of clinical application determines, to a great extent, the bioprosthesis availability referred to as an organ from the shelf. Several data sets have indicated that the storage temperature may affect the tissue morphology and biomechanics, but the data are limited to the study of native allografts or homografts and do not explain what can occur in acellular tissue during cold storage. They also do not explain how the storage conditions of explanted tissues can affect the acellularization process. Only a small number of publications describe issues related to pulmonary valve biomechanics. In a paper of Mookhoek the biomechanics of failed and normal pulmonary autografts was compared. They observed that the mechanical response of failed pulmonary autografts was nonlinear, which is typical for the healthly human arterial tissue [27]. In the paper of Seebacher, the native or decellularized porcine pulmonary heart valve conduits with or without cryopreservation was compared with human cryopreserved pulmonary valve conduits. It was observed that the porcine pulmonary heart valve showed the higher failure force after cryopreservation. The porcine cryopreserved porcine conduits showed a higher extensibility [28]. In the study of Christie, the mechanical properties of porcine pulmonary and aortic valve leaflets was compared. When fresh the pulmonary valve leaflets showed higher extensibility and they were less stiff [29]. Studies on the impact of storage conditions on biomechanics and morphology are described for other tissues than pulmonary valves, therefore, it is necessary to extrapolate the results obtained for other collagen tissues. In the study of Chow [30], the bovine thoracic aorta was stored under different conditions at 4, −20, and −80 °C for 48 h, 1 week and 3 weeks, and the biaxial tensile test and collagen content assay were performed. They found the decrease in the initial slope and increase in the stiff slope after 48 h when stored at 4 °C. However, the storage at −20 and −80 °C caused an increase in the stiff slope that did not affect the initial slope. These changes were combined with a decrease in the soluble collagen content during storage. The study suggests better maintenance of biomechanics during freezing compared with refrigeration. The fracture problem associated with the cryopreservation of the aorta was investigated by Hu [31], and the impact of temperature, freezing rate, and cryoprotective agent on the mechanical properties of rabbit aorta and fracture modes was assessed. At −20 °C, typical ductile fracture was observed; after lowering the temperature to −50 and −80 °C, typical brittle fracture appeared. The crack growth is reduced as the temperature decreases. The effect of freezing on the passive mechanical properties of arterial samples was studied by Delgadillo [32] using samples differentiated based on the type of freezing medium (isotonic saline solution, Krebs-Henseleit buffer solution with dimethyl sulphoxide (DMSO) and storage temperature. A portion of the samples after freezing were stored at −20 °C, and the remainder was stored at −80 °C; after 2 months, the samples were thawed and mechanically tested. Well-preserved biomechanical properties were obtained using Krebs solution with DMSO (at −20 or at −80 °C) or isotonic saline solution at −20 °C. It was concluded by the authors that the method of tissue storage determines the biomechanical properties. Therefore, the study of tissue biomechanics is burdened with error when the sample is not properly stored since harvesting up to testing or bioprosthesis preparation. Most biomechanical studies are performed on tissue harvested within days after animal death and not on the fresh tissues, assuming that the storage at low temperature and use of suitable media allow the biomechanics to remain unchanged [33]. In the study of Blondel [34], higher stiffening of femoral aorta samples was observed in the samples cryopreserved at −80 and −150 °C than in fresh aorta samples. Differences exist between the rheological properties of fresh and cryopreserved aorta. In contrast to these studies, the results reported by Adham [35] indicate no significant differences in biomechanical properties of fresh aorta samples and cryopreserved ones using DMSO. Therefore, there is a need to underline some parameters that are useful to compare the mechanical behaviour of tissue stored under different conditions. In the study of Venkatasubramanian [36], the pig femoral aorta was frozen in the presence of cryoprotective agent (CPA) at −80 °C and in the absence of CPA at −20 °C. To test the biomechanical properties, the uniaxial tensile test was performed. The results obtained by the authors indicate that freezing affects the stress–strain properties of the aorta, particularly in the low-stress region. One of the possible mechanisms of the observed changes may be damage to the ECM. Another aspect of the effect of freezing on the mechanical stability of tissues was presented in the paper of Xu [37] in which the thermal behaviour of rabbit aorta during freezing was analysed. The effect of different concentrations of the dimethyl sulphoxide (DMSO) cryoprotective agent and different freezing rates on the thermal expansion was investigated indicating strong correlations among the DMSO concentration, freezing rate, and biomechanical stability. It was postulated that the cracks observed in freezing tissue are due to thermal expansion. In our studies, the examined tissue showed macroscopic homogeneity, and there were no significant differences in tissue thickness. The tissues were taken from animals of similar weight and age. Thus, we minimised the possible influence of the morphological parameters on the tested biomechanical properties. It was observed that in the case of tissues that were subjected to deep-freezing and then stored for 1 week in −140 °C followed by acellularization (G4) and in the tissues in which the acellularization was performed before deep-freezing and storage in liquid nitrogen (G5), the examined valves showed higher stiffness than the control and 4 °C stored tissue samples. This phenomenon was especially evident in the G4 group. There was a significantly higher value of the modulus of elasticity, energy to break, peak load, and peak stress within this group. At the same time, lower values of the peak strain were observed in this group, further indicating the greater stiffness of these tissues. It should be highlighted that the observed changes were more pronounced for the tissues examined in the circumferential direction. It could be attributed to the anisotropic structure of collagen tissue. The observed changes in the biomechanical properties of the tissues may be related to their morphological changes. Comparing the group of tissues stored in 4 °C with that stored in liquid nitrogen, no significant qualitative changes were observed. The tissues exhibited similar intensity of staining. It seems, however, that deep-frozen tissues are characterised by a greater compactness of ECM fibres. It may be associated with changes in fibre alignment caused by ice crystal formation during the freezing process. Additionally, water movement can influence the microstructure of the ECM. In the frozen sample, the fibres could be already aligned, and the tissue increased in stress more rapidly than fresh tissue. This type of data underlined how the changes in water redistribution and ice crystal formation may affect the mechanical behaviour of the examined tissue [36]. The data were also consistent with the results obtained by Elder [38] indicating that, because of the anisotropic structure, collagen is mainly oriented in the circumferential direction and collagen during freezing develops higher stiffness than that in the axial direction. The latter finding is related to the so-called thermal cross-linking of collagen [39] fibres and explains why, after thawing collagen, the matrix might be reinforced in the circumferential direction, making it stiffer. Observation of the changes in biomechanics can also be useful to predict possible haemodynamic changes. This is due to the Moens–Korteweg equation. It is assumed that the flow is pulsating, and the arteries is a cylindrical tube with elastic walls.

$$c = \sqrt {\frac{{Eh}}{{{\mathrm{\rho a}}}}}$$

*c*-wave speed of the blood flow, *E*-Elastic modulus of the arterial wall, *h*-arterial wall thickness, *ρ*-density of blood, *a*-internal diameter of the artery.

Several studies indicate that due to the increase in the elastic modulus, the wave speed is increased in frozen tissue [40]. The changes in mechanical properties during freezing can also influence the haemodynamic conditions and induce plaque formation [41]. The influence of biomechanical properties on the porcine aortic and pulmonary conduits was also the subject of our previous study in which the finite element method (FEM) was used to estimate the influence of the biomechanical properties on the haemodynamic conditions. We demonstrated that the increase in the modulus of elasticity affected the Velocity Stream Line, Wall Shear Stress, and Turbulence Eddy Dissipation [16]. An alternative to storing tissues in liquid nitrogen is refrigerated storage at + 4 °C that is used both for short- and long-term periods to avoid tissue decomposition. In the study of Durong [42], the effect of storage at + 4 °C on the mechanical properties of the porcine liver and spleen was examined. The effect was compared with fresh and freeze-thaw cycle samples. The authors observed the decrease in the mechanical properties of the spleen that is explained by the autolysis of elastin by elastolytic enzymes during refrigeration. In was observed by Stemper [43] that porcine arterial tissue stored at + 4 °C for 24 and 48 h present changes in mechanical properties with the decrease in elastic modulus. Those effects could be related to tissue degeneration during cold storage. Opposite results were obtained by Ikeyama [44]; the increase in elasticity of rat lungs was observed after 9 h of storage in a refrigerator. In turn, in the research conducted by Amin [45], 1–28 days storage of the carotid artery at + 4 °C resulted in changes in mechanical properties. Long-term storage causes the degeneration of elastin fibres, leading to changes in stress and stretch. In our study, we observed that, for tissue stored at + 4 °C for 1 week and then subjected to the acellularization procedure (G3 group), there was a decrease in the modulus of elasticity compared with that in the control sample and G1 and G2 groups. Similar relationships were reported for the Energy to Break value, Peak Load, and Peak Stress. Additionally, the peak strain was approximately 20% lower in the G3 group than in the control. These changes were especially pronounced in relation to the samples tested in the axial direction. While in Slope 1, the initial elastic region of the stress–strain curve, indicates the measured values were similar to those in the control and G1 and G2 group, in Slope 2, which indicates the stiff region of the stress–strain curve, the storage of tissue for 1 week in + 4 °C prior to acellularization resulted in a decrease in the measured value. Changes in biomechanical parameters seem to be reflected in the changes in tissue morphology. In the case of elastin, EVG staining showed no significant differences between the groups. Hence, there was no difference in the initial elastic region of the stress–strain curve (Slope 1). In turn, fibronectin, collagen I, and collagen IV staining indicates a higher density of tissue in the G3 group, particularly for the samples tested in the circumferential direction. This can be reflected by the lower value of the stiff region of the stress–strain curve (Slope 2) registered for G3 group. Additionally, the changes in tissue morphology, especially the observed higher density, may affect the lower efficiency of acellularization in this group. These results coincide with the literature data. In the study of Menard [46], the mechanical properties of the growth plate were tested to determine the optimal storage condition. The samples were stored at + 4 °C for periods of 1, 2, 3, and 6 days and were compared with those stored at – 20 °C. It was concluded that cold storage for more than 48 h alters the biomechanics of tissue, primarily the maximal stress, equilibrium stress, matrix modulus, and permeability. Sancho [47] presented the results of the effect of extended cold storage on the stability of collagen and collagen-agarose hydrogels. It was observed that the storage modulus and loss of modulus of collagen gels were decreased during extended (30 days) cold storage, the values were increased for collagen-agarose gels. In the study of Ghoson [48], the stability of porcine aortic tissue at + 4 °C after 10 days of storage was estimated. The research included the examination of tissue permeability and evaluation of tissue morphology. After 10 days, the permeability for glucose increased by nearly 300%, and similar morphological changes were observed, as indicated by the increase in the average pore area. The research carried out indicates that the optimal storage condition for maintaining the biomechanical stability and appropriate morphological properties of the scaffold intended for the bioprosthesis construction based on the tissue-engineered techniques, is storage at 4 ^o^C. This particularly applies to the group of tissues subjected to acellularization immediately after the antibiotic bath, and those that have been subjected to acellularisation and after that has been stored at 4 °C. This has been confirmed both in the biomechanical and morphological study. Tissue properties in these groups were similar to those in native tissue which was used as a control.

## Conclusions

Tissue-engineering of the heart valve appears to be a highly promising method that can be used in the cardiac surgery of heart valve replacement. However, despite considerable progress in this area, this method is still not widely used clinically. There are no exact standards for the preparation of heart valve bioprosthesis. This method’s application among other methods of tissue acellularization is dedicated to the preparation of acellular scaffolds, the methods of cell seeding on the scaffolds and selection of proper cell source. An important issue seems to be the choice of storage conditions of the tissue. Proper selection of storage methods can affect the morphological properties and biomechanical stability of the biological scaffold. The understanding of the effect of storage conditions on the nature of changes in the scaffold morphology and biomechanics is crucial and of important clinical significance, both for the postoperative and long-term results. Therefore, in our study, we tested the influence of different storage conditions on the morphology and biomechanics of pulmonary heart valves. For all the samples, we performed the static tensile test and examined the viscoelastic properties and changes in ECM proteins. It was observed that the storage conditions can significantly influence the biomechanics and morphology of valve prostheses that, in turn, can affect the haemodynamics of bioprostheses and their durability. Based on our study, it seems that storage of acellular tissues at 4 °C allows for maintaining optimal biomechanical and morphological properties of the scaffolds intended for the use of bioprosthesis construction using the tissue-engineering techniques. The properties of such tissues are similar to native tissues. To the best of our knowledge, these are the first studies that comprehensively address these issues, and the storage conditions of scaffolds of tissue-engineered heart valves may have a significant impact on the clinical effect of the used bioprostheses and can contribute to establishing standards in this area.
